# Reorganized Genomic Taxonomy of *Francisellaceae* Enables Design of Robust Environmental PCR Assays for Detection of *Francisella tularensis*

**DOI:** 10.3390/microorganisms9010146

**Published:** 2021-01-11

**Authors:** Caroline Öhrman, Jason W. Sahl, Andreas Sjödin, Ingrid Uneklint, Rebecca Ballard, Linda Karlsson, Ryelan F. McDonough, David Sundell, Kathleen Soria, Stina Bäckman, Kitty Chase, Björn Brindefalk, Shanmuga Sozhamannan, Adriana Vallesi, Emil Hägglund, Jose Gustavo Ramirez-Paredes, Johanna Thelaus, Duncan Colquhoun, Kerstin Myrtennäs, Dawn Birdsell, Anders Johansson, David M. Wagner, Mats Forsman

**Affiliations:** 1 CBRN Defence and Security, Swedish Defence Research Agency, FOI, SE 901 82 Umeå, Sweden; caroline.ohrman@foi.se (C.Ö.); andreas.sjodin@foi.se (A.S.); perssoningrid@hotmail.com (I.U.); linda.karlsson@foi.se (L.K.); david.sundell@foi.se (D.S.); stina.backman@foi.se (S.B.); bjorn.brindefalk@foi.se (B.B.); emil.hagglund@icm.uu.se (E.H.); johanna.thelaus@foi.se (J.T.); kerstin.myrtennas@foi.se (K.M.); 2 Pathogen and Microbiome Institute, Northern Arizona University, Flagstaff, AZ 86011, USA; Jason.Sahl@nau.edu (J.W.S.); rgb65@nau.edu (R.B.); Ryelan.McDonough@nau.edu (R.F.M.); Kathleen.Soria@nau.edu (K.S.); Dawn.Birdsell@nau.edu (D.B.); Dave.Wagner@nau.edu (D.M.W.); 3 US Army Medical Research Institute, Fort Detrick, MD 21702, USA; kitty.chase@dhhs.nc.gov; 4 Logistics Management Institute supporting Defense Biological Product Assurance Office (DBPAO) Joint Project Lead, CBRND Enabling Biotechnologies (JPL CBRND EB), Frederick, MD 21702, USA; shanmuga.sozhamannan.ctr@mail.mil; 5 School of Biosciences and Veterinary Medicine, University of Camerino, 62032 Camerino, Italy; adriana.vallesi@unicam.it; 6 Ridgeway Biologicals Limited a Ceva Santé Animale Company, Units 1-3 Old Station Business Park, Compton, Berkshire, England RG20 6NE, UK; gus.ramirez@ceva.com; 7 Fish Health Research Group, Norwegian Veterinary Institute, Oslo, Pb 750 Sentrum, 23 N-0106 Oslo, Norway; duncan.colquhoun@vetinst.no; 8 Department of Clinical Microbiology, Clinical Bacteriology, and Laboratory for Molecular Infection Medicine Sweden (MIMS), Umeå University, SE-901 85 Umeå, Sweden; anders.f.johansson@umu.se

**Keywords:** *Francisella* taxonomy, tularemia, phylogeny, assay

## Abstract

In recent years, an increasing diversity of species has been recognized within the family *Francisellaceae*. Unfortunately, novel isolates are sometimes misnamed in initial publications or multiple sources propose different nomenclature for genetically highly similar isolates. Thus, unstructured and occasionally incorrect information can lead to confusion in the scientific community. Historically, detection of *Francisella tularensis* in environmental samples has been challenging due to the considerable and unknown genetic diversity within the family, which can result in false positive results. We have assembled a comprehensive collection of genome sequences representing most known *Francisellaceae* species/strains and restructured them according to a taxonomy that is based on phylogenetic structure. From this structured dataset, we identified a small number of genomic regions unique to *F. tularensis* that are putatively suitable for specific detection of this pathogen in environmental samples. We designed and validated specific PCR assays based on these genetic regions that can be used for the detection of *F. tularensis* in environmental samples, such as water and air filters.

## 1. Introduction

The family *Francisellaceae* consists of a group of Gram-negative, non-motile, aerobic bacteria with diverse life cycles. In recent years, this family has been expanded vastly and new genera as well as many new species have been included and characterized. The family is currently comprised of three genera: *Francisella*, *Allofrancisella* [1], and *Pseudofrancisella* [2]. The genus *Pseudofrancisella* contains a single validly published species isolated from estuarine seawater [2]. The genus *Allofrancisella* contains three validly published species, of which all have been isolated from water samples collected from cooling towers of air conditioning systems [1]. Despite this diversity within the *Francisellaceae,* and nine different *Francisella* species (Table A1), only the three *F. tularensis* subspecies, i.e., subspecies *tularensis* (type A), subspecies *holarctica* (type B), and subspecies *mediasiatica* [3], are of significant clinical relevance to humans. The subspecies *holarctica* and *tularensis* cause tularemia in humans [4]. Knowledge relating to the virulence of *F. tularensis* subspecies *mediasiatica* in humans, which is primarily found in Central Asia, is limited; however, an intermediate position between the other two subspecies in terms of virulence is assumed [5,6]. *F. tularensis* subspecies *tularensis* is highly infectious in humans and a number of other mammalian species, and is most feared for its potential as a biological weapon [7] or bioterrorism agent [8].

*F. noatunensis*, *F. orientalis, F. marina* [9] and *F. halioticida* [10] are the etiological agents of francisellosis [11,12,13] in cold water fish species, warm water fish species, and mollusks, respectively [10,14]. The genus *Francisella* also includes opportunistic pathogens that can cause disease in immunocompromised humans (i.e., *F. novicida*, *F. hispaniensis*, and *F. philomiragia*) [4,15,16,17,18,19]. In addition, the genus *Francisella* includes *Francisella*-like endosymbionts (FLE) of ticks and ciliates (i.e., *F. persica*, *F. endociliophora*, and *F. adeliensis*) [20,21,22]. Related ‘environmental’ taxa, including *F. salina*, *F. salimarina*, *F. uliginis*, *F. frigiditurris*, *Allofrancisella* spp., and *Pseudofrancisella aestuarii* [1,2,23,24,25,26,27,28,29,30], have been isolated or sequenced directly from samples of brackish water, sea water, water from air conditioning systems, cooling towers, and/or other environmental sources, including natural warm springs or ice machines, suggesting that *Francisellaceae* inhabit a wide diversity of ecological niches. This group includes both opportunistic pathogens and non-pathogenic species that may become aerosolized from air-cooling systems and also be captured on air filters [1,31,32]. Of note, the BioWatch program in the USA, which tests for the presence of pathogenic organisms on air filters, reported 149 false positive results between 2003 and 2014, of which the majority were associated with molecular assays for *F. tularensis* [32]. Given the ubiquity of diverse *Francisella* species in the environment, and the fact that many of them can apparently become aerosolized, it seems likely that the presence of these other species on air filters are the source of these false positive BioWatch results.

There are multiple natural sources of transmission of *F. tularensis* to humans including contaminated water and food; direct contact with infected animals such as rabbits, hares, squirrels, voles and other rodents; and bites from ticks, flies and mosquitoes or inhalation of aerosol [33]. However, the relative frequency of the different modes of transmission of *F. tularensis* to humans vary according to the geographic areas considered and local ecology. *F. tularensis* is well known for its environmental persistence and irregularity of outbreaks, sometimes separated by several years, that can occur in closely confined geographical regions (aka foci). The principal natural reservoir is largely unknown, although studies have shown that the organism may persist for more than 16 months in water or mud [34]. Hence, from a surveillance perspective, vectors and complex air, water, and sediment samples are all relevant. Analysis of vectors and complex environmental samples is challenging in relation to distinguishing the human disease-causing *F. tularensis* from genetically similar species that may also be present in the samples.

Many *Francisella* species are difficult or impossible to culture directly from the environment, which has limited our understanding of this genus. This is even true for *F. tularensis*, where cultivation of the agent from the environment has traditionally been performed by inoculation of environmental samples into laboratory animals, because direct isolation by culturing of environmental samples is commonly compromised by overgrowth of other bacterial species on most growth media. In recent years, *F. tularensis* (type B) was isolated directly from drinking water in Turkey by filtering samples of spring water through cellulose acetate membranes before cultivation on selective media [35]; however, this seems to be a rare exception. The FLEs, especially, are notoriously difficult to cultivate directly from their hosts, although there are some examples of successful isolations [22]. In addition, advances in culture-independent approaches and direct DNA sequencing of complex environmental samples have revealed an extensive diversity of *Francisella* near-neighbor species [36,37,38]. Thus, many more environmental *Francisella* species likely exist that remain to be identified and explored.

*F. tularensis* is a very monomorphic ISspecies with a very small accessory genome [39,40], possibly because its evolutionary history entailed a transition from a free-living bacterium to a host-associated pathogen [41]. The *F. tularensis* genome consists of a single circular chromosome of about 1.9 million base pairs with a very high AT content (~68%) [42]. Differences between *F. tularensis* and other *Francisellaceae* species that are primarily isolated from environmental sources include metabolic competence, which is higher among the environmental species, and signs of ongoing genome erosion in *F. tularensis* that has resulted in the deletion of metabolic pathway components (i.e., genes involved in amino acid biosynthesis) [41,42]. *F. tularensis* is also characterized by a marked increase in insertion sequences [39]; large numbers of insertion sequences are often associated with an evolutionary process resulting in the loss of gene functionality. Interestingly, the etiological agent of francisellosis in cold water fish, *F. noatunensis*, also contains large numbers of insertion sequences that are similar to those reported for *F. tularensis*, and the high degree of clonality that characterizes *F. tularensis* [41] is also found in *F. orientalis* [39]. Despite infecting different species (fish vs. mammals), it appears that these pathogenic lineages share certain important evolutionary features. The chromosome of *F. tularensis* has a duplicated *Francisella* pathogenicity island (FPI). In contrast, the genome of opportunistic *F. novicida* contains a single FPI. *F. tularensis* harbors a degenerated CRISPR/Cas system [43], whereas the more metabolically versatile *F. novicida* possesses a well-functioning Type II CRISPR/Cas system [44]. The primary role for CRISPR/Cas is in defense against invading foreign nucleic acids, a function that thus seems to be lost in *F. tularensis*. This might suggest a life cycle with little competition and/or association with other microorganisms. In support of this notion, there is an absence of recombination in *F. tularensis* strains, especially compared with *F. novicida*, which has a significant rate of recombination [39].

To further elucidate the genomic differences between the non-disease-causing relatives, or near-neighbors, within the family *Francisellaceae*, and the human disease-causing *F. tularensis*, we have analyzed a dataset comprising 499 whole-genome sequences, including 172 environmental *F. tularensis* near-neighbors and 327 *F. tularensis* genome sequences. We identified the very few genomic regions unique to *F. tularensis* and demonstrated the utility of specific PCR assays based on these unique regions for the robust detection and identification of *F. tularensis*.

## 2. Materials and Methods

### 2.1. Library Preparation

DNA libraries were generated at Northern Arizona University (NAU), the Swedish Defense Research agency (FOI), and SciLifeLabs in Uppsala, Sweden. At NAU, DNA Libraries were prepared using the KAPA Library Preparation Kits with SRPI Solution and Standard PCR Library Amplification/Illumina series (KAPA Biosystems, Code KK8232) using the following modifications: adapters and 8 bp index oligos from IDT^®^ (Integrated DNA Technologies, San Diego, CA, USA) based on Kozarewa and Turner, 2011 [45], were used in place of those supplied in the KAPA preparation kit. The DNA sample (~1 µg) was fragmented using a SonicMan (Matrical) and fragment targets of 600–650 bp were selected using Agencourt AMPure XP beads (Beckman Coulter, Code A63882). At FOI, DNA libraries were prepared from 1 ng DNA following the Nextera^®^ XT protocol (Illumina, San Diego, CA, USA). At SciLifeLabs, 35 DNA libraries were prepared from 1 µg DNA using TruSeq DNA sample prep kit v2 (Illumina, cat# FC-121-2001/2002) following the manufacturer’s instructions.

### 2.2. Genome Sequencing

In total, 217 isolates were sequenced with Illumina sequencing technologies using MiSeq, HiSeq2000, or HiSeq2500 instruments. MiSeq sequencing was performed using the 500-cycle MiSeq Reagent Kit V2 or the 600-cycle MiSeq Reagent Kit V3 at FOI or NAU following the reagent protocol. DNA libraries prepared with TruSeq kits were run on HiSeq sequencing platform located at SciLife. Sequencing was performed with paired end 200-cycles with V3 Reagents. For each sample, information about the number of cycles and instrument platform is available at SRA (see accession numbers in Table S1).

### 2.3. Genome Assembly

Samples sequenced at FOI were assembled using abyss-pe 2.2.2 [46] using an k-value of 51. Contigs shorter than 500 bp were removed and the assembly was polished using pilon v1.22 [47]. The assemblies were checked for contamination with kraken2 v2.07 using a kraken-standard database (all available genomes from Archaea, Bacteria, human, UniVec_Core and Viral). Samples sequenced at NAU were adapter-trimmed with bbduk v38.86 (https://sourceforge.net/projects/bbmap/) and assembled with SPAdes v3.13.1 [48].

### 2.4. Genome Sequences

The complete dataset used for analysis consisted of 499 genome sequences; 217 newly sequenced and 282 downloaded from NCBI (Table S1). Six additional genomes (GCF_003428165.1, GCF_003574425.1, GCF_002803295.2, GCF_003428155.1, GCF_003574485.1, GCA_006227905.1), reclassified as part of the *Francisellaceae* family by Genome Taxonomy Database (GTDB Release 05-RS95, 17th July 2020) [49], and *Piscirickettsia salmonis* LF 89 (GCF_000297215.2), which is genetically similar to *Francisellaceae* [39], were also included in the initial phylogenetic tree. Recommended representative genome assemblies are listed in Table S4.

### 2.5. Phylogenetic Trees

**Figure 1:**
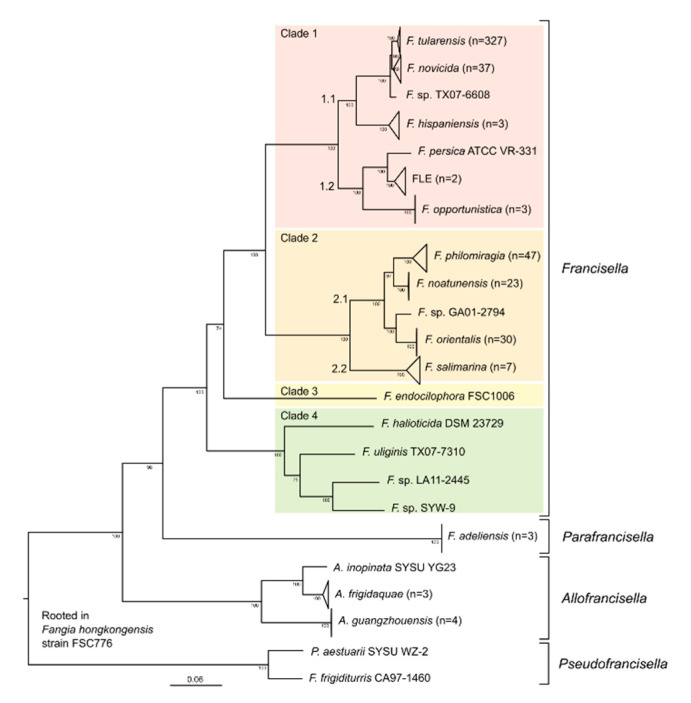
Whole genome maximum likelihood phylogeny of the complete *Francisella* near neighbor (FNN) dataset calculated with RAxML. Genus *Francisella* is divided into four separate clades indicated with different colors and numbered 1–4. The four most proximate genera to *Francisella* are also presented (the complete phylogenetic tree of *Francisellaceae* is presented in Figure A1).

Phylogenies were made by first aligning each genome to the reference strain SCHU S4 (GCA_000008985.1) using progressive mauve (snapshot_2015_02_13) [50] with default settings, converting them into the FASTA format according to the reference coordinate system, and merging them into one multi FASTA. All SNPs were then extracted using SNP-sites (v2.51) keeping only ACTG sites [51]. Ten starting trees were calculated using RAxML-HPC-PTHREADS-SSE3 [52] with model GTRGAMMA (--no-bfgs) and 100 bootstraps were calculated for the best ML tree using the same model. From the bootstrap replicate tree, bipartitions on the best maximum likelihood tree were drawn using model GTRCAT. The tree was visualized in FigTree 1.4.3 and rooted on *Fangia hongkongensis* for the complete dataset (Figure 1), *Francisella novicida* strain U112 (CP000439.1) for the clade 1 subset (Figure 3), and *Piscirickettsia salmonis* LF 89 GCF_000297215.2 for *Francisellaceace* (Figure A1).

### 2.6. Average Nucleotide Identity

Average nucleotide identity (ANI) was calculated for the dataset consisting of 499 genomes using pyani v0.2.10 [53] and the ANIb (blast) method. The correlation table was aggregated per species by mean first on the x-axis and then on the y-axis using the aggregate function in the R package stats. The resulting table was visualized using function heatmap.2 from the R package ggplot2 (v3.1).

### 2.7. Genome Taxonomy of Francisellaceae

Information downloaded from Genome Taxonomy Database (GTDB) was incorporated into the phylogenetic tree together with ANI values. The taxonomy from GTDB was compared to the original NCBI taxonomy assignment and type strains not yet included in GTDB were used to update names of unassigned species in the GTDB backbone.

### 2.8. Identification of Clade-Specific Regions

For each genome assembly, coding region sequences (CDSs) were predicted with Prodigal v2.63 [54]; intergenic regions > 50 nucleotides were also extracted from genome assemblies. All regions were concatenated and clustered with cd-hit v4.8.1 [55] at an identification threshold of 90%. Each representative sequence from each CDS was aligned against all queried genomes with BLAST v2.9.0+ [56] and the blast score ratio (BSR) [57] was calculated. The BSR value is calculated by dividing the query alignment bit score by the reference alignment bit score; a BSR value of 0.8 is approximate to 80% identity over 100% of the alignment length. CDSs unique to each group were identified by the compare_BSR.py script; this requires that a unique region has a BSR value of ≥0.9 in all target genomes and a BSR value of <0.4 in all non-target genomes. The visualization of unique regions was performed by iTOL v5.6.1 [58].

### 2.9. Design of TaqMan qPCR Assays

Two of the unique *F. tularensis* regions were of a sufficient length (>300 nts) and associated with coding regions (FTS_0772, FTS_1286). Primers were designed to these targets with Primer3 v2.3.6 [59]; the specificity of primer sequences was determined through the in silico PCR tool, VIPR v0.0.1 (https://github.com/TGenNorth/vipr). The primer sequences were evaluated against NCBI BLAST databases (nr/nt and wgs) to check for potential cross-amplification in other isolated and uncultivated bacteria.

### 2.10. In Silico Evaluation of Francisella Primers

Previously published primer/probe sets for *F. tularensis* detection were processed across all analyzed genomes (*n* = 499) in this study using VIPR. Sensitivity and specificity were calculated using *F. tularensis* as the target species (Table S2). A subset of the highest scoring assays was selected, and targeted genome regions were extracted from the FNN dataset and aligned to evaluate the theoretical performance and uniqueness of these assays.

### 2.11. Validation of Francisella tularensis Specific Assays

We tested three potential *F. tularensis*-specific TaqMan assays on a diverse panel of *Francisella* DNA samples to validate their specificity to *F. tularensis*. Two of these assays were previously published [60,61] and a third assay was developed in this study (Table 1). The latter assay (Ft-sp.FTS_0772) targets a *F. tularensis*-specific signature identified in this study through in silico analysis of diverse *Francisella* genomes (Table S1). This novel assay was designed as a TaqMan real-time PCR assay using Primer Express software (Thermo Fisher). A minor groove binding moiety was attached to the short probe to increase the probe’s melting temperature and stabilize probe-target hybridization. Each TaqMan real-time PCR assay was run with a 10 µL reaction volume and comprised of the following components: 1× TaqMan Universal PCR master mix (Life Technologies, Applied Biosystems, Foster City, CA), primers (Integrated DNA Technologies, San Diego, CA, USA) and probe (Life Technologies, Applied Biosystems, Foster City, CA) (Table 1), and 1 μL of DNA template (~1 ng). These assays were run on Life Technologies Quant Studio Instruments Flex Real-Time PCR System under conditions described (Table 1) using the following thermal cycle conditions: 50 °C for 2 min, 95 °C for 10 min, and 50 cycles of 95 °C for 15 s and 60 or 63 °C for 1 min (Table 1). Each qPCR experiment included two negative controls and two positive control DNA samples per assay. The sensitivity of the PCR reactions (Ft-sp.FTT0376, Ft-sp.3Pan and Ft-sp.FTS_0772) was evaluated using dilution series with concentrations of *F. tularensis* DNA between 10 ng and 1 fg.

### 2.12. Rationale for Francisella Panel Selection

We selected 84 *Francisellaceae* DNA species/strains (Table S1) to test the specificity of three potential *F. tularensis*-specific TaqMan assays (Table 1), including 40 *F. tularensis* and 44 non *Francisella tularensis* that were selected based on the diversity (genetic and geographic) and availability of DNA samples. Representative strains were included for most groups (Figure 1 and Table 5), but a few groups were not represented due to a lack of availability of DNA samples. The *Francisellaceae* panel included strains characterized with whole genome sequencing (*n* = 65) as well as isolates for which whole genome sequences were not available (*n* = 19) (Table S3). The unsequenced isolates were genetically assigned to species using previously published genotyping methods [62]. To demonstrate that all DNAs included in the panel supported PCR, we first screened the panel using an assay that targets the bacterial 16S rRNA gene [63].

## 3. Results

### 3.1. Organisation of Genera and Species Based on Whole-Genome Analysis

Based on the GTDB definition [49], *Francisellaceae* includes a total of nine genera divided into three major branches (Figure A1). The dataset included in this study covers the entire clade in which the genera *Francisella, Parafrancisella*, *Allofrancisella*, and *Pseuodfrancisella* are located (with addition of the selected outgroup genome of *Fangia hongkongensis*) and is therefore regarded as the “*Francisella* near neighbor” (FNN) dataset (Figure 1). The remaining four genera within the *Francisellaceae* are *Caedibacter*, represented with three genomes, and the three genera with single sequenced representatives: *Fastidiosibacter*, QLIT01 (a currently unnamed genus), and M0027 (another currently unnamed genus) [49].

The genus *Francisella* consists of 11 different species according to the GTDB definition. The classification of *F. novicida* as a separate species or subspecies of *F. tularensis* has been debated [19,64,65]. The recognized species *F. philomiragia* and *F. noatunensis* have been considered synonymous in GTDB, and according to their classification, *F. noatunensis* should rather be considered as a subspecies of *F. philomiragia*. However, as illustrated in Figure 1, *F. noatunensis* represents a monophyletic sister clade population to *F. philomiragia*. *F. orientalis* and GA01-2794 are also regarded as a single species, which correlates with ANI values of 95%.

### 3.2. Phylogenetic Tree

In the phylogenetic tree (Figure 1), the genus *Francisella* is divided into four separate clades (clades 1–4) consistent with results obtained from GTDB analysis. Clades 1 and 2 have been previously described [39]. In clade 1, the *Francisella* isolate TX07-6608 [66] is closely related to *F. novicida* and *F. tularensis* within the sub-clade 1.1. In sub-clade 1.2, considerable diversity is identified among FLEs, and *F. opportunistica* is a new addition. In clade 2, *F. salimarina* is a new addition and comprises sub-clade 2.2, whereas sub-clade 2.1 includes *F. philomiragia*, *F. noatunensis*, *F. orientalis,* and the new candidate species GA01-2794. Clade 3 contains the single genome of *F. endociliophora* as the only representative. Clade 4 consists of recently discovered taxa, each of which represents a novel species. The candidate genus *Parafrancisella* is represented by the single species *Parafrancisella adeliensis.* The genus *Allofrancisella* consists of three species, *A. inopinata, A. frigidaquae*, and *A. guangzhouensis*, whereas the genus *Pseudofrancisella* consists of two species: *P. aestuarii* and *F. frigiditurris*. Based on the extended *Francisellaceae* dataset (Figure A1), the order between *Parafrancisella* and *Allofrancisella* is uncertain.

### 3.3. Average Nucleotide Identity

**Figure 2:**
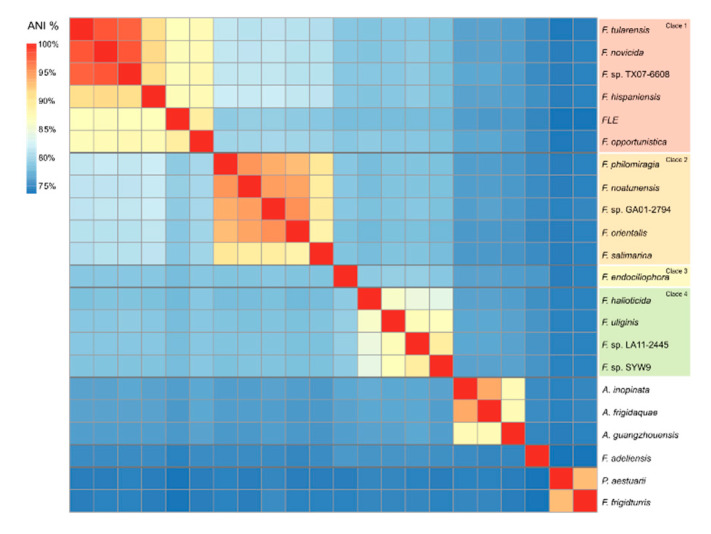
Clustered heatmap visualizing ANI values between *Francisella*, *Allofrancisella*, *Parafrancisella*, and *Pseudofrancisella* species.

ANI values ranged from 73.6% between *Parafrancisella adeliensis* and *Pseudofrancisella aestuarii* to 98.1% between *F. tularensis* and *F. novicida* (Figure 2). The mean inter-clade ANI values varied among clades 1–4, with clade 1 most similar to clade 2 (78.7–81.8%) and least similar to clade 4 (77.7–78.9). The intra-clade ANI value varied between 83.8% (clade 4) and 98.1% (clade 1), with the lowest value of 77.6% observed between FLE and *P. aestuarii* SYW9. Mean ANI values between *Francisella* and the other genera (*Allofrancisella*, *Parafrancisella* and *Pseudofrancisella*) varied between 73.8% and 76.9%.

### 3.4. Subspecies and Major Genotypes within Francisella tularensis

**Figure 3:**
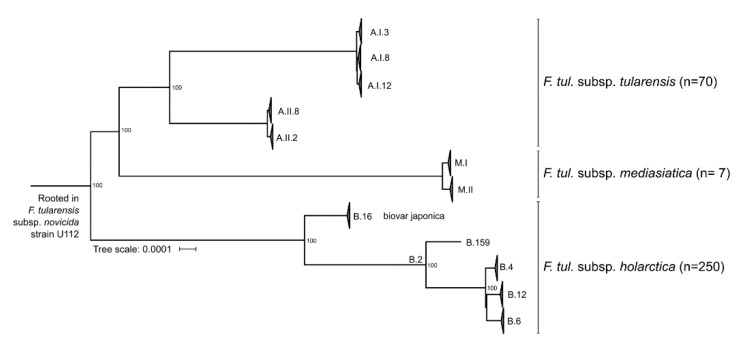
Phylogenetic tree of *F. tularensis* visualizing subspecies and major genetic subgroups.

*Francisella tularensis* is divided into three subspecies: *tularensis*, *mediasiatica*, and *holarctica* (Figure 3); *F. novicida* is regarded as a separate *Francisella* species and not a subspecies of *F. tularensis*. *F. tularensis* subspecies *tularensis* (Type A) is further separated into two distinct genetic groups, A.I and A.II, with three major branches in A.I [67,68] and two major branches in A.II [69,70]. *F. tularensis* subspecies *mediasiatica* is divided into clades, M.I and M.II, wherein M.II is represented by three recently available genomes from Russia [71]. A third clade, M.III, has been proposed but no genomes are available for this clade [5]. *F. tularensis* subspecies *holarctica* (Type B) is divided into two major branches B.16 and B.2, wherein the latter is further divided into the four branches B.4, B.6, B.12, and B.159 according to canSNP nomenclature [72]. Most of the known isolates of *F. tularensis* subspecies *holarctica* belong to the three branches B.4, B.6, and B.12 [73,74,75,76]. Branch B.16 is usually named “biovar japonica” after where it was first discovered The clade with canSNP designation B.159 is represented by a single strain, F0835, to date only described from California, USA [69]. This strain, phylogenetically assigned to clade B.2 (Figure 3), falls between B.16 and the other three major clades in the *F. tularensis* subspecies *tularensis* (B.12, B.6 and B.4) phylogeny.

### 3.5. Francisella Tularensis Specific Genomic Regions

A total of six unique genomic regions were identified within the *F. tularensis* core genome that were absent in all near neighbor species, based upon a BSR value ≥ 0.9 in all *F. tularensis* genomes and a BSR value < 0.4 in all near-neighbor genomes (Table 2). Annotation of these regions, performed using Prodigal, could not match the full coding regions with regions annotated in GenBank; the specific coordinates for each unique region are, however, reported across two reference genomes (Table 3).

### 3.6. Genomic Regions Specific to Individual Species

To identify species-specific genomic regions, a comparative BSR analysis was performed. The results identify genomic regions unique for each clade (Table 2). The pan genome of fish pathogens (*F. orientalis* and *F. noatunensis*) includes the lowest number of unique gene variants, whereas *F. novicida* and *F. philomiragia* includes the largest number of unique gene variants.

### 3.7. Genomic Regions Specific to Francisella Tularensis Subspecies and Major Phylogentic Groups

**Figure 4:**
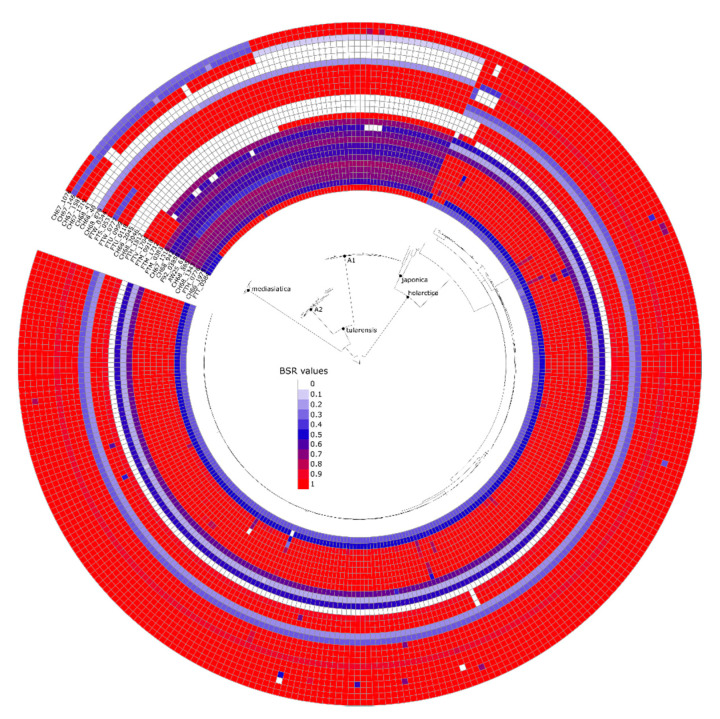
Visualization of genomic regions that are differentially present/absent across the three subspecies of *F. tularensis* and/or major phylogenetic groups within the subspecies. A phylogeny is presented in the middle of the figure, and the multiple circles around the phylogeny correspond to different genomic regions. The color of each cell in the circles corresponds to the BSR value for each genomic component in each examined whole genome sequence.

We also attempted to identify genomic regions unique to each of the three *F. tularensis* subspecies, as well as major phylogenetic groups within those subspecies. In this analysis, we did not implement a hard threshold for gene absence but, rather, searched for consistently more conserved regions between groups. The results demonstrate variable conservation across the three subspecies (Figure 4, Table 4), suggesting differentiation of these subspecies over time. Evolution in *F. tularensis* is mainly driven by gene loss [77], so the presence of genes unique to a particular subspecies could be associated with the persistence of ancestral regions in one subspecies that were subsequently lost in the other subspecies.

### 3.8. In Silico Evaluation of PCR Assay Specificity

PCR primers were screened against all genome assemblies using VIPR, an in silico PCR tool. The number of positive calls was determined by the identification of a predicted amplicon size. A limitation of this approach is that VIPR requires exact matches of primers and probe. In practice, a single nucleotide mismatch may still work in the laboratory. Sensitivity and specificity were calculated using the assumption that assays should only target *F. tularensis* and miss all near neighbors, including the closely related *F. novicida*. The results demonstrate that only four assays (Ft-sp.FTT0376, Ft-sp.3Pan, Ft-sp.FTT0523, and Ft-sp.FTS_0772) demonstrated 100% sensitivity and specificity towards *F. tularensis* (Supplementary Table S3), including the assay designed in this study. The PCR primers designed in this study show no cross-amplification when evaluated against sequences from other bacteria.

The recently published Ft-sp.3Pan assay [61] targets a genomic region that is not unique to *F. tularensis*. Thus, the specificity of this assay to *F. tularensis* is based upon utilization of primers designed for a portion of this region that differs between *F. tularensis* and near-neighbor species by the presence of several SNPs. Of note, the targeted region is closely located to a mobile element (ISFtu1), which could complicate assay evaluation via in silico analyses, especially when using incomplete, unclosed genomes. At least four non-*F. tularensis* genomes in the FNN dataset contain the targeted region. These genomes (two *F. philomiragia*, one *F. novicida*, and one *F. salimarina*), collectively, have one mismatch in the forward primer, three mismatches in the reverse primer, and one mismatch in the probe sequence. The Ft-spFTT0376 assay [60] targets a region in *F.* sp. TX07-6608 with conserved primer regions that could potentially cause positive results. Many other *Francisellaceae* species have alignments to this region but there are no perfect primer matches, so it is unclear if any of these species could cause positive results with this assay. The genomic target of assay Ft-sp.FTT0523 [60] is shared between *F. tularensis* and some strains of *F. novicida*, with, collectively, two mismatches in the forward primer, three mismatches in the reverse primer, and five mismatches in probe sequences. It should be noted that only *F. tularensis* subspecies *tularensis* subtype A1 has no mismatches in the probe sequence of this assay. *F. tularensis* subspecies *holarctica*, *F. tularensis* subspecies *mediasiatica*, and *F. tularensis* subspecies subtype A2 contain one mismatch in the probe sequence.

### 3.9. Validation of Francisella tularensis Specific Assays

The three potential *F. tularensis*-specific TaqMan assays all robustly amplified *F. tularensis* DNA but varied in exclusion of near-neighbors (Table 5). Two assays (Ft-sp.FTT0376 and Ft-sp.FTS_0772) were highly specific to *F. tularensis*. Among 84 diverse strains in the *Francisellaceae* DNA panel, all 40 *F. tularensis* DNAs yielded positive results, whereas all 44 near-neighbor strains yielded negative results with these two assays. These results indicate that both of these assays are both highly sensitive and specific for *F. tularensis*. The Ft-sp.3pan assay was also very sensitive, yielding positive results for the 40 *F. tularensis* DNAs, but demonstrated less specificity compared to the other two assays as indicated by robust amplification of DNA from two *F. philomiragia* near-neighbor strains (Table 5). The failure of this assay on these two near-neighbor DNA samples was not due to poor DNA quality, as they yielded robust amplification with 16S qPCR. Taken together, these data support that two assays, Ft-sp.FTS_0772 and Ft-sp.FTT0376, are highly sensitive and specific to *F. tularensis* (Table 5). The sensitivity of the three evaluated PCR reactions (Ft-sp.FTT0376, Ft-sp.3Pan and Ft-sp.FTS_0772) are between 1 and 10 fg.

## 4. Discussion

This study represents the most comprehensive description to date of genomic taxonomy and diversity for the family *Francisellaceae*. We analyzed a whole genome dataset consisting of 327 *F. tularensis* genomes and 172 non-*F. tularensis Francisellaceae* genomes. We recognized nine genera within the family *Francisellaceae*, including four in the *Francisella* near neighbor (FNN) dataset (*Francisella, Allofrancisella, Pseudofrancisella*, and *Parafrancisella)*. Based on genetic distances, the genus *Francisella* is best divided into four major clades (Figure 1). These new data were used to increase and validate the specificity of DNA-based assays to detect *F. tularensis.* A genomic region unique to *F. tularensis* was used to design a novel PCR assay (Ft-sp.FTS_0772) that, against a diagnostic validation panel as well as the in silico testing represented by diversity within the FNN dataset, demonstrated superior or equivalent specificity compared to previously published assays.

The genus *Francisella* was previously split into two main genetic clades [39], corresponding to clades 1 and 2 in this study. Briefly, *F. tularensis* strains, which can infect mammals, were assigned to clade 1, whereas *F. noatunensis* and *F. orientalis* strains, which can infect fish, were assigned to clade 2 [39]. In the expanded FNN genome dataset reported here, four major clades are recognized within genus *Francisella*, but it should be noted that the clade division of strains infecting mammals as clade 1 and strains infecting fish as clade 2 still remains valid, although more diversity have been added to both clades. A recent global phylogenetic analysis of the genus *Francisella* based on 63 genomes [40] divided the genus into three major clades (A-C), roughly corresponding to clades 1, 2, and 4 presented here. However, the rooting used in this analysis resulted in *F. persica*, *F. opportunistica*, and *F. hispaniensis* not being assigned to any major clade, and this analysis did not include all of the species and genera included in this current study.

In the four-clade phylogenetic structure for *Francisella* presented in this study, clade 1 contains *F. tularensis* together with *F. hispaniensis* in one sub-clade and the FLEs and *F. opportunistic* in the other sub-clade. In general, the majority of clade 1 strains are either endosymbionts (FLEs) of ticks, or facultative intracellular pathogens almost exclusively isolated from humans or other warm-blooded animals. The FLEs only replicate intracellularly and can be transmitted transovarially [38,78,79]. Unlike *F. tularensis*, FLEs do not grow in cell-free media and their transmission to and virulence in humans is unknown [80,81]. However, the single isolate of *F. persica* seems to be a transitional form in terms of ability to grow on cell-free media, since it can be cultivated on agar plates but grows extremely slowly (14–20 days). On the other hand, not all members of clade 1 originate from mammals or vectors. Exceptions include a new member of clade 1, *Francisella* isolate TX07-6608, that was directly isolated from seawater [66] and the first isolate of *F. novicida*, an exceedingly rare opportunistic human pathogen, that was directly isolated also from salt water [15]. Clearly, *F. novicida* show distinct differences to *F. tularensis* in clinical, ecological, genomic and virulence properties [82]. This, in combination with the genome clustering of the 37 strains of *F. novicida* shown in this study, supports maintaining *F. novicida* and *F. tularensis* as separate species.

Although none of the three human pathogenic *F. tularensis* subspecies have been directly associated with seawater, this capability is present in other members of clade 1. A rare case of *F. hispaniensis* infection was acquired from seawater [28]. Likewise, the first reported *Francisella* strain isolated from the Southern Hemisphere originated from a patient infected in brackish water in Australia (*F. hispaniensis* strain 3523) [39,83]. Interestingly, it has been shown that *F. tularensis* subspecies *tularensis* shows enhanced survival in brackish water, suggesting that salt-influenced (marine) environments may promote its survival [84]. Together, these findings are in agreement with a previous suggestion that *F. tularensis* may have evolved in and from the marine environment [42].

Clade 2 contains the fish pathogens *F. noatunensis* and *F. orientalis*, as well as *F. philomiragia* and *F. salimarina*. Strains of *F*. *philomiragia* cause disease primarily in immunocompromised hosts and/or salt-water near-drowning victims [18,85]. All known strains of *F. salimarina* have been directly isolated from seawater and, as such, their potential clinical relevance is as yet unknown [86]. Although very rare, the majority of human infections caused by non-*F. tularensis* members of clade 1 (*F. novicida*, *F. hispaniensis)* and some members of clade 2 (*F. philomiragia)* tend to occur in immunocompromised individuals following exposure to salt or brackish water [87].

Clade 3 is represented by a single isolate of *F. endociliophora,* an endocytobiont of the marine ciliate species *Euplotes* [88]. *F. endociliophora* was directly isolated and cultured in cell free media at room temperature from *E. raikovi*, a temperate water ciliate species [20]. Another endocytobiont of *Euplotes*, *P. adeliensis*, the only isolate of the candidate genus *Parafrancisella*, was isolated in a similar manner from *E. petzi* [21]. It is well established that the virulent *F. tularensis* subspecies are deficient in biosynthesis pathways for many amino acids, a trait that is shared by both these endocytobionts (*F. endociliophora* and *P. adeliensis*), suggesting a shared host dependency for supply of amino acids. This further strengthens the assumption of a marine origin for *F. tularensis*. Strains that constitute clade 4 in the present dataset (this study) are all non-clinical isolates, including isolates from seawater, cooling water systems, and abalone [23,26]. Thus, clade 4 contains what appears to be free-living bacteria in the environment.

Within the dataset analyzed (499 genomes), we identified only six short sequence regions apparently unique to *F. tularensis.* We used a strict definition for a region to be called unique, which may have limited the comprehensive identification of unique signatures. Another explanation why there are so few regions unique to the virulent *F. tularensis* is that the evolution of *F. tularensis* is characterized by genome erosion (Figure 4), so that rare unique sequence regions are remnants that have persisted through the evolution of *F. tularensis*, which suggest that these regions may play an important role to the lifecycle of *F. tularensis*. Two of the regions are intergenic and three are partial loci of either pseudogenes or hypothetical proteins. The 6th, region 4, constitutes a partial domain of the adenosine deaminase gene (FTT 0939), a gene encoding a key enzyme in purine metabolism, but this gene is predicted to be functional only in *F. tularensis* subspecies *tularensis* [89,90]. Most bacterial pathogens causing systemic infections seems to lack a functional adenosine deaminase gene, an absence that is probably compensated by host metabolic pathways [91]. The reason for the presence of an apparently intact adenosine deaminase gene only in *F. tularensis* subspecies *tularensis* is unknown.

The main concern with *F. tularensis*-specific assays is not false-negatives, as *F. tularensis* is well characterized and highly monomorphic. Rather, the challenge is the unknown diversity that is assumed to exist amongst uncultivable bacterial species and especially environmental members within clade 1 that remain to be explored, as indicated by the recently characterized *F. opportunistica* and *F. hispaniensis*-like strains (Figure 1). Region 1 is the target for the new PCR assay developed in this study (Ft-sp.FTS_0772) that is highly specific for *F. tularensis*. This unique sequence region is located in the terminal remnant of the *hsdS* gene and originally encoded a type 1 restriction enzyme, congruent with the gene erosion, resulting in the significant loss of restriction capability in virulent *F. tularensis* [92]. We also screened previously published PCR assay primers that were developed for specific identification of *F. tularensis* against all genome assemblies in our dataset. Only two assays, Ft-sp3Pan [61] and Ft-sp.FTT0376 [60], demonstrated high sensitivity and specificity for *F. tularensis* in these in silico analyses. However, a more detailed alignment showed theoretical specificity issues with both of these assays. It should be noted that these alignments per se would not disqualify these assays; high specificity could in practice still be obtained. Therefore, these two assays were included, as well as the assay designed in this study, in the wet-bench validation against the *Francisella* strain diversity panel. The results reveal that two of the assays, Ft-sp.FTS_0772 (this study) and Ft-sp.FTT0376 [60], are both highly sensitive and specific to *F. tularensis*. A caveat in this validation was, however, the lack of strain TX07-6608 in the panel. This taxon could, at least theoretically, compromise the specificity of the Ft-sp.FTT0376 assay. Another finding was that the Ft-sp 3pan assay did not exclude all near-neighbors on our diversity panel, as two *F. philomiragia* strains (F1091 and F1093) amplified robustly. In silico analysis revealed that strains F1091 and F1093 were the only examples of the tested 23 *F. philomiragia* strains in the wet-bench validation panel that harbor the target genome region of the Ft-sp 3pan assay. The targeted region is closely located to a mobile element (ISFtu1), which may explain the absence of this region in many of the strains. Taken together, we would rather recommend using the PCR assay designed in this study, or Ft-sp.FTT0376, for detection of *F. tularensis* in environmental and other complex samples.

This study dramatically expands the genome space of *Francisellaceae.* Previous diagnostics for *F. tularensis* have suffered from a largely shared but unexplored *Francisellaceae* pan genome, resulting in false positive results, which is problematic for routine biothreat surveillance. The unique signatures identified in this study may also be shared by unexplored, unculturable, and previously undetected *Francisella* near neighbor species that have yet to be characterized. Continued culture-independent characterization of these cryptic species will expand our understanding of the diversity of these lineages.

## Figures and Tables

**Table 1:** Primers and TagMan-MGB probes for real-time assays targeting *F. tularensis*.

Assay	Region in Genome of SCHU S4 ^a^	Sequences of Forward and Reverse Primers ^b^ and Probes ^c^	Primer (µM) ^d^	MGB ^e^	Probe (µM) ^f^	Tm °C ^g^
Ft-sp. FTT0376	377718–377824	F CCATATCACTGGCTTTGCTAGACTAGT R TGTTGGCAAAAGCTAAAGAGTCTAAA FAM-AAATTATAAAACCAAACCCAGACCTTCAAACCACA	0.9	no	0.2	60
Ft-sp.3Pan	8967–9049	F TTTACACCCGTCTCCGTTAGT R CTCTTAAGGATGCAATTTGGGATT FAM-AAGAGGCAAAGCTGGAATTACACTCTCTC	0.9	no	0.2	60
Ft-sp. FTS_0772	1187646–1187807	F CAAGGTAAAGAAATTAAGAGTAGTAAAGTTGAATTC R ATTTAATCTAGTATTATCAATTGGGTAAAAAGGTA FAM-GATGTGGCAACAACTGAAAT	0.9	yes	0.2	63

^a^ Position coordinates of assay target within reference SchuS4 whole genome sequence (AJ749949.2); ^b^ F: forward primer, R: reverse primer; ^c^ 6-carboxyfluorescein (FAM) fluorophore and a 3= black hole quencher-1 (BHQ-1) quencher; ^d^ Primer concentration (μM) per reaction; ^e^ Minor Groove Binding moiety on probe; ^f^ Probe concentration (μM) per reaction; ^g^ Real-time PCR annealing temperature.

**Table 2:** Overview of genome distribution within clades and species, including detailed information about the number of unique regions for each species and pan/core genome size.

Genus	Clade	Species	#Genomes	#genomes	#Unique Regions	Pan-Genome Size (#Genes)	Core-Genome Size (#Genes)
*Francisella*	clade 1	*F. tularensis*	374	327	6	2401	1541
		*F. novicida*		37	2	2949	1499
		*F.* sp. TX07-6608		1	250	N/A	N/A
		*F. hispaniensis*		3	121	2015	1599
		FLE and *F. persica*		3	170	2293	1006
		*F. opportunistica*		3	464	1751	1713
	clade 2	*F. philomiragia*	113	47	10	2992	1611
		*F. noatunensis*		23	74	1944	1766
		*F.* sp. GA01-2794		1	180	N/A	N/A
		*F. orientalis*		30	36	2287	2025
		*F. salimarina*		7	191	2371	1786
	clade 3	*F. endociliophora*	1	1	1340	N/A	N/A
	clade 4	*F. halioticida*	4	1	1190	N/A	N/A
		*F. uliginis*		1	736	N/A	N/A
		*F.* sp. LA11-2445		1	587	N/A	N/A
		*F*. sp. SYW 9		1	1179	N/A	N/A
*Parafrancisella*		*F. adeliensis*	3	3	1874	1936	1925
*Allofrancisella*		*A. inopinata*	7	1	377	N/A	N/A
		*A. frigidaquae*		2	211	1479	1356
		*A. guangzhouensis*		4	715	1570	1455
*Pseudofrancisella*		*P. aestuarii*	2	1	375	N/A	N/A
		*F. frigiditurris*		1	406	N/A	N/A

**Table 3:** Detailed information regarding the six genomic regions unique to *F. tularensis* based upon in silico analyses.

Region	LVS * Coordinates	SCHU S4 ** Coordinates	Marker Length	Annotation	Notes
1	1490829–1491161	1187496–1187828	333	hsdS, type I restriction modification domain protein	partial locus
2	84841–85406	378437–377872	567	FTT_0376c, hypothetical membrane protein	partial locus
3	1673808–1673858	547774–547824	51	FTT_0525, conserved hypothetical protein	
4	43283–43397	951193–951307	114	add1, adenosine deaminase	partial locus
5	43766–43874	950716–950824	109	intergenic, region between bioA and add1	
6	135869–136156	765604–765891	288	FTT_0742, hypothetical protein	partial locus

* CP009694.1, ** GCA_000008985.1.

**Table 4:** Number of genomic regions that are unique to or have been lost across the three subspecies of *F. tularensis* and/or major phylogenetic groups within the subspecies.

Species	Subspecies	Genetic Group	#Unique Regions	#Regions Lost	Number of *F. tularensis* Subspecies Region is Present in
*F. tularensis*	*tularensis*	A	70	1	3
		A1	39	0	2
		A2	31	0	2
	*holarctica*	B (excluding japonica)	250	0	0
		japonica	5	1	2
		B including japonica	255	7	2
	*mediasiatica*	M	7	3	2

**Table 5:** Assay performance for isolates included in the validation panel. Number of isolates indicates the number of strains per species/subspecies included in the validation panel. Numbers in the other columns indicate the number of positive results (i.e., purported *F. tularensis*-positive results) for each of the three examined PCR assays.

	#Isolates	Ft-sp.FTT0376	Ft-sp.3Pan	Ft-sp.FTS_0772
*Francisella tularensis* subspecies *tularensis*	25	25	25	25
*Francisella tularensis* subspecies *holarctica*	13	13	13	13
*Francisella tularensis* subspecies *mediasiatica*	2	2	2	2
*Francisella novicida*	7			
*Francisella hispaniensis*	2			
*Francisella persica*	1			
*Francisella FLE*	1			
*Francisella opportunistica*	1			
*Francisella philomiragia*	23		2	
*Francisella halioticida*	1			
*Francisella noatunensis*	1			
*Francisella orientalis*	1			
*Francisella salimarina*	1			
*Francisella endociliophora*	1			
*Parafrancisella adeliensis*	1			
*Allofrancisella inopinata*	1			
*Allofrancisella frigidaquae*	1			
*Allofrancisella guangzhouensis*	1			

**Figure A1:**
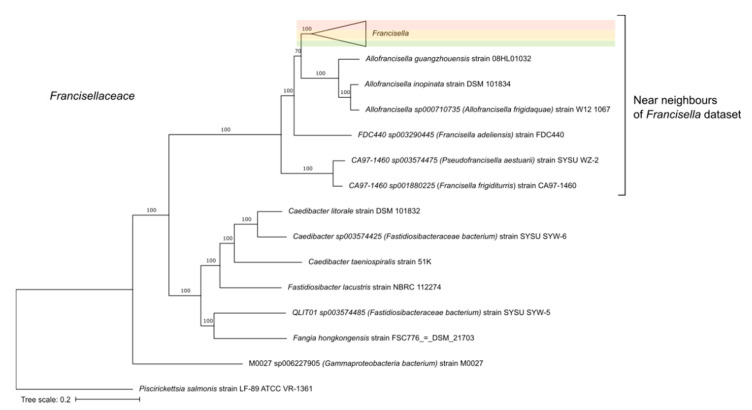
Phylogenetic tree of family *Francisellaceae*.

## Data Availability

The data generated in this study are openly available in NCBI project accession PRJNA657836. Public available genome data presented in this study is listed in Supplementary Table S1.

## Supplementary Materials

The following are available online at https://www.mdpi.com/2076-2607/9/1/146/s1, Table S1: Metadata describing included *Francisellaceae* isolates, Table S2: In silico validation of PCR assays, Table S3: Metadata and performance of validation panel, Table S4: Recommended genome assemblies for *Francisellaceae* species and subspecies.

## Appendix A

**Table A1:** Detailed overview of species within family *Francisellaceae* following NCBI taxonomy.

*Francisellaceae* genus	*Francisellaceae* species	Heterotypic Synonyms	Type Strains	References
*Francisella*	*Francisella tularensis*			[93]
	*Francisella tularensis* subspecies *tularensis*		SchuS4	[17,42,94,95]
	*Francisella tularensis* subspecies *holarctica*	*Francisella tularensis var. palaearctica*	FSC257; GIEM 503	[17,95,96]
			FSC200		
	*Francisella tularensis* subspecies *mediasiatica*		FSC147	[41,95,97,98]
	*Francisella tularensis* subspecies *novicida*	*F. novicida, Pasteurella novicida*	ATCC 15482; CCUG 33449; CIP 56.12	[15,17,19]
			U112		
	*Francisella hispaniensis*		CCUG 58020; DSM 22475; F62; FhSp1; FnSp1; FSC454	[19]
	*Francisella persica* (FLE)	*Wolbachia persica*	ATCC VR-331; DSM 101678; FSC845	[22,99,100,101]
	*Francisella opportunistica*		ATCC BAA-2974; DSM 107100; PA05-1188	[23,102,103]
	*Francisella philomiragia*	*F. philomiragia* subspecies *philomiragia, Yersinia philomiragia*	ATCC 25015; CCUG 19700; CCUG 4992; CIP 82.98; DSM 7535	[18,82,104]
	*Francisella noatunensis*	*F. piscicida; F. philomiragia* subspecies *noatunensis Francisella noatunensis* subspecies *noatunensis,*	2005/50/F292-6; DSM 23596; LMG 23800; NCIMB 14265	[11,12,13,105]
	*Francisella orientalis*	*F. victoria, F. asiatica, Francisella noatunensis* subspecies *orientalis*	DSM 21254; Ehime-1; LMG 24544	[11,12,13]
			CCUG 56120T; DSM 21294; PQ 1104		
	*Francisella salina*		TX07-7308	[23,66]
	*Francisella marina*		E103-15, E95-16	[9]
	*Francisella salimarina*		CGMCC 1.17031; NBRC 113781; SYSU SYW-1	[83]
	*Francisella endoc* *iliophora*	*F. noatunensis* subspecies *endociliophora*		[20,86]
	*Francisella halioticida*		DSM 23729; LMG 26062; Shimane-1	[10,26]
	*Francisella* sp. LA11-2445		LA11-2445	[25]
	*Francisella uliginis*		TX07-7310	[23,66]
	*Francisella adeliensis*		FSC1327	[21]
	*Francisella frigiditurris*		CA97-1460	[23]
*Allofrancisella*	*Allofrancisella inopinata*		DSM 101834; KCTC 42968; SYSU 23; SYSU YG23	[1,27]
	*Allofrancisella frigidaquae*		DSM 101835; KCTC 42969; SYSU 10HL1970	[1,24,27]
	*Allofrancisella guanzhousensis*	*F. guanzhousensis, F. cantonensis*	08HL01032; ATCC BAA-2361; CCUG 60119; NCTC 13503	[1,31,106]
*Pseudofrancisella*	*Pseudofrancisella aestuarii*		CGMCC 1.13718; KCTC 52557; SYSU WZ-2	[2]
